# Physical and Pharmacological Restraints in Hospital Care: Protocol for a Systematic Review

**DOI:** 10.3389/fpsyt.2019.00921

**Published:** 2020-02-28

**Authors:** Wendy de Bruijn, Joost G. Daams, Florian J. G. van Hunnik, Arend J. Arends, A. M. Boelens, Ellen M. Bosnak, Julie Meerveld, Ben Roelands, Barbara C. van Munster, Bas Verwey, Martijn Figee, Sophia E. de Rooij, Roel J. T. Mocking

**Affiliations:** ^1^Department of Psychiatry, Amsterdam UMC, University of Amsterdam, Amsterdam, Netherlands; ^2^Medical Library, Amsterdam UMC, University of Amsterdam, Amsterdam, Netherlands; ^3^Verpleegkundigen & Verzorgenden (V&VN), Utrecht, Netherlands; ^4^Dutch Geriatric Society (NVKG), Utrecht, Netherlands; ^5^Department of Geriatrics, UMCG, Groningen, Netherlands; ^6^Amsterdam UMC, University of Amsterdam, Amsterdam, Netherlands; ^7^Alzheimer Nederland, Amersfoort, Netherlands; ^8^Stichting Mind, Amersfoort, Netherlands; ^9^Department of Internal Medicine/Geriatrics, Gelre Hospitals and UMCG, Groningen, Netherlands; ^10^Department of Hospital Psychiatry, NVvP, Utrecht, Netherlands; ^11^Department of Psychiatry, Icahn Medical School at Mount Sinai, New York, NY, United States; ^12^Department of Elderly Medicine, UMCG, Groningen, Netherlands

**Keywords:** physical restraint, pharmacological restraint, chemical restraint, hospital, adverse effects, complications, behavioral issues, systematic review protocol

## Abstract

**Background:**

Physical and pharmacological restraints, defined as all measures limiting a person in his or her freedom, are extensively used to handle unsafe or problematic behavior in hospital care. There are increasing concerns as to the extent with which these restraints are being used in hospitals, and whether their benefits outweigh their potential harm. There is currently no comprehensive literature overview on the beneficial and/or adverse effects of the use of physical and pharmacological restraints in the hospital setting.

**Methods:**

A systematic review of the existing literature will be performed on the beneficial and/or adverse effects of physical and pharmacological restraints in the hospital setting. Relevant databases will be systematically searched. A dedicated search strategy was composed. A visualization of similarities (VOS) analysis was used to further specify the search. Observational studies, and if available, randomized controlled trials reporting on beneficial and/or adverse effects of physical and/or pharmacological restraints in the general hospital setting will be included. Data from included articles will be extracted and analyzed. If the data is suitable for quantitative analysis, meta-analysis will be applied.

**Discussion:**

This review will provide data on the beneficial and/or adverse effects of the use of physical and pharmacological restraints in hospital care. With this review we aim to guide health professionals by providing a critique of the available evidence regarding their choice to either apply or withhold from using restraints. A limitation of the current review will be that we will not specifically address ethical aspects of restraint use. Nevertheless, the outcomes of our systematic review can be used in the composition of a multidisciplinary guideline. Furthermore, our systematic review might determine knowledge gaps in the evidence, and recommendations on how to target these gaps with future research.

**Systematic Review Registration:**

PROSPERO registration number: CRD42019116186.

## Introduction

### Rationale

Physical and pharmacological restraints are still being used extensively by health professionals in general hospital care (1–4). Physical and pharmacological restraints can be defined as measures that limit a person in his or her freedom (5). Physical restraints are any action or procedure that prevents a person’s free body movement to a position of choice and/or normal access to his/her body by the use of any method, attached or adjacent to a person’s body that he/she cannot control or remove easily (4, 6). Examples of physical restraints range from applying the brakes on a wheelchair or raising the bed rails, to using an abdominal restraint (5, 7–9). Even though considered less invasive, methods such as using video, mechanical (e.g., seat exit alarms), or acoustical surveillance are also seen as restraints (10). Pharmacological, also called chemical, restraints are a form of restraint in which drugs are used to restrain patients. Although no consensus definition exists, they can be defined as either the deliberate or incidental use of pharmaceutical products to control a person’s behavior and/or to restrict his or her freedom of movement, when they are not exclusively intended to treat a medical condition (8, 11). Medicaments such as benzodiazepines and antipsychotics, that are commonly used in psychiatric practice, for example in, respectively, the treatment of insomnia and the treatment of psychosis, are also used as pharmacological restraints due to their sedative effects. Physical and pharmacological restraints are mostly used when a patient shows behavior that compromises his or her own safety and that can cause serious physical or mental injuries, or compromises the safety of others (4, 12). Examples of this behavior range from wandering and making repetitive or disturbing noises to agitated and aggressive behavior or even suicidality (13, 14). If used properly, restraints are only used in cases where there is no alternative and less invasive measure possible. Moreover, only the least invasive or restraining measure that is effective in a given situation should be used (5, 9).

In the last few years, medical, ethical, and political concerns have increasingly risen about the extent with which restraints are used in hospitals and whether their beneficial effects outweigh their potential harm (1–4, 15). Potential harm associated with restraints is numerous, for example, malnutrition, bed sores, incontinence, contractures, falling, as well as mental deterioration and worsening of the behavior that was the reason to use the restraint in the first place (5, 9, 16). The use of restraints is also known to have a negative psychological impact on patients, provoking feelings of fear or anger, as well as feelings of embarrassment and the experience of loss of dignity (2, 16). Furthermore, the evidence on the benefits of restraints is not convincing, e.g., restraints do not always seem to be effective in reducing falls, or in preventing patients from removing their medical devices (4, 17, 18). A majority of the patients admitted to hospitals nowadays are elderly. Elderly patients are one of the patient groups with a higher risk to experience physical or pharmacological restraints (9). Furthermore, they are also more prone to suffer from the adverse consequences of these restraints than younger patients. Restraints may even increase the chance of physical or mental harm, instead of reducing it, particularly in the elderly (9). Considering the potential harm and the fact that the evidence suggests that restraints are not unequivocally effective in preventing the harmful situations they were indicated for in the first place, the question is whether restraint use is ethically justifiable. Moreover, restraints always imply a far-reaching restriction of personal freedom and are often applied in situations where patients are not able to give permission for the intervention themselves, and where one has to rely on permission from a guardian (19, 20). In the Netherlands, these concerns have gained attention in the political setting and have recently led to the development of a new quality indicator on the use of restraints and an update of the law on coercion in care (15, 21). In some other countries, e.g., the United Kingdom and Iceland, the use of restraints is even already restricted or prohibited in certain settings (22).

Currently, there is no comprehensive literature overview on the beneficial and/or adverse effects of the use of physical and pharmacological restraints in the hospital setting. Therefore, health professionals that are being confronted with unsafe or problematic behavior lack evidence based guidance. Hospitals in the Netherlands have constructed their own guidelines, but there is no national or international consensus on how and when to use physical or pharmacological restraints in hospitals. Importantly, there are signs that the hospitals’ own guidelines are not always used appropriately when applying restraints. In Dutch research from 2015, only 31% of 346 interviewed nurses followed a guideline while using a restraint on a patient (3). In a Belgian study, only 26.9% of participating physicians used guidelines for the pharmacotherapeutic management of agitation (23). Furthermore, it was recently shown that most of the nurses participating in a study did not understand the reasons for restraint use (24). The lack of available evidence based guidance may cause uncertainty in the practice of applying restraints and wrongful use of restraints.

In conclusion, there seems to be a gap between new developments and increasing attention for reducing restraints in hospitals on the one hand, and a lack of evidence based practice for using restraints on the other hand. In this project, we aim to address this gap by systematically reviewing the available literature on the beneficial and/or adverse effects of the use of physical and pharmacological restraints in the hospital setting.

### Objectives

Our main objective is to systematically accumulate and critically review the available evidence on the beneficial and/or adverse effects of different physical and pharmacological restraints in the hospital setting. We intend to do this by collecting evidence on different outcomes that report information on either a beneficial or an adverse effect of the use of restraints. For example, both a shorter length of hospital stay for restrained patients, as well as a shorter time for a patient to reach a state of tranquillity after administration of a pharmacological restraint implicate a beneficial effect of the use of restraint. In contrast, a high rate of complications (e.g., falls, adverse drug events, or agitation) occurring in patients that were in restraints during their hospital stay implicates an adverse effect of the use of restraints.

## Methods

### Review Method

This systematic review protocol was drafted according to the preferred reporting items for systematic review and meta-analysis protocols (PRISMA-P) 2015 statement (25). The preferred reporting items for systematic review and meta-analysis (PRISMA) checklist will be used throughout the process of drawing up the systematic review (26). The protocol is registered in the PROSPERO international prospective register of systematic reviews.

### Eligibility Criteria

In this section, we specify the criteria by which we will select the studies that will be included in the systematic review.

The first criterion for inclusion we are considering is the study design. We will be including observational studies, e.g., prospective and retrospective cohort studies, case-control studies, cross-sectional studies, case series with sample sizes equal to or larger than 10, as well as experimental studies if available, e.g., randomized controlled trials. By surveying relevant literature, we expect that most available studies will be observational studies, while experimental studies are not expected to be widely available.

Another criterion we will be selecting studies on is the setting the studies are conducted in. Participants in studies should be admitted to the hospital. We define the hospital setting as all medical wards of general hospitals, including but not limited to surgical or geriatric wards, and emergency departments of general hospitals. Studies including patients admitted to intensive care units (ICU) will be excluded considering an ongoing review of another research group specifically on that subject (27). Moreover, we exclude this group considering it is a different population admitted in distinct conditions, which are not generalizable to other wards of a general hospital. Also studies conducted on psychiatric wards will be excluded, given that these studies address a specific patient group and care setting that does not generalize to the general hospital. Studies not conducted in general hospitals will be excluded. Consequently, we will exclude studies conducted in nursing homes and other long term care facilities, as well as studies conducted in psychiatric institutions or psychiatric hospitals. We exclude these settings considering the already available evidence on the subject in these settings (28–30). Studies that contain data from general hospitals as well as nursing homes or other care facilities will be used if they clearly separate the results for each setting, or if these results can be provided separately by the authors of these studies.

The next selecting criterion is the intervention that studies look in to. The intervention we are interested in is the use of physical or pharmacological restraints. These restraints have to be used with the intention to aid the safety of the patient and its surroundings, for example, when a patient shows behavior that compromises his or her own safety or the safety of others and that can cause serious physical or mental injury. We will not include studies using physical or pharmacological interventions that are applied exclusively with the intention to treat a disease or disorder, e.g., antipsychotics prescribed with the intention to treat a delirium, restricting or forcing dietary intake in case of, e.g., refeeding, or benzodiazepines prescribed with the intention to treat sleeping disorders or alcohol withdrawal. The comparators we are interested in are either no use of physical or pharmacological restraints, use of alternative measures instead of physical or pharmacological restraints, or use of less invasive or restraining physical or pharmacological restraints. Specifically, if the literature allows, pharmacological restraints are compared either to a placebo or to other pharmacological restraints, e.g., antipsychotics compared to benzodiazepines or a placebo. For physical restraints, a multitude of different comparisons can be made. For example, comparisons between standard restraints and less invasive restraints such as bed- or seat exit alarms, motion detectors, or acoustical surveillance. Furthermore, comparisons between standard restraints and new, possibly safer and more effective restraints, such as safe enclosures. Another possibility is a comparison between access to a bed- or seat exit alarm versus no access to such an alarm. Also expected to be available are studies looking into associations between restraint and other factors, such as delirium or falls. For example, such a study could investigate the association of restraint in a group of patients suffering from delirium compared to a group of patients without delirium.

Another important criterion for selection of studies are the study outcomes. To provide a structured overview of our outcomes of interest, we listed our outcomes of interest in **Table 1**. A further elaboration on the outcomes is described below in the *Outcomes and Prioritization* section.

**Table 1:** Outcomes of interest.

Primary outcomes			
Length of hospital stay			
Complication rate	Overall		
	Divided by type of complication	Physical health	Falls
			Walking dependence
			ADL dependence
			Pressure ulcers
			Contractures
			Strangulation
			Laceration
			Other
		Mental health	Cognitive performance
			Behavioral issues
			Depressive symptoms
			Anxiety
			Agitation
			Aggression
			Other
Success rate	Overall		
	Specific measures	Time to tranquility or sedation	
		Need for additional sedation	
**Secondary outcomes**			
Survival			
Symptom severity	Somatic		
	Psychiatric		
Effect on the healthcare system	Healthcare providers (e.g., nurses, physicians)		
	Health costs		
	Cost effectiveness		

Other study characteristics we will take into account while conducting the selection process are timing, geographical setting, and language. We will include articles published from inception to the present date. We will include studies in the English and Dutch language. If relevant studies in other languages are available, they can be used, providing that there is a usable translation. There are no restrictions for the geographical locations the studies are conducted in. We will include studies that have been fully published.

### Information Sources

The information sources we will use are the MEDLINE, Embase, and PsycINFO databases. In addition, we will scan reference lists of included studies and relevant guidelines to ensure that no relevant studies will be excluded from the review.

### Search Strategy

We have consulted an experienced medical information specialist for composing the search strategy. Firstly, we performed a scoping search in Google Scholar, including backward and forward reference citation analyses and similar article searches in PubMed. This yielded reference sets on the subjects of physical restraints and pharmacological restraints.

To further optimize the search, we composed a comprehensive list of physical restraints and problem behavior types and synonyms using related guidelines, overview articles, and a consultation of a clinical neuropsychologist (7, 9, 13, 14, 31–38).

**Figure 1:**
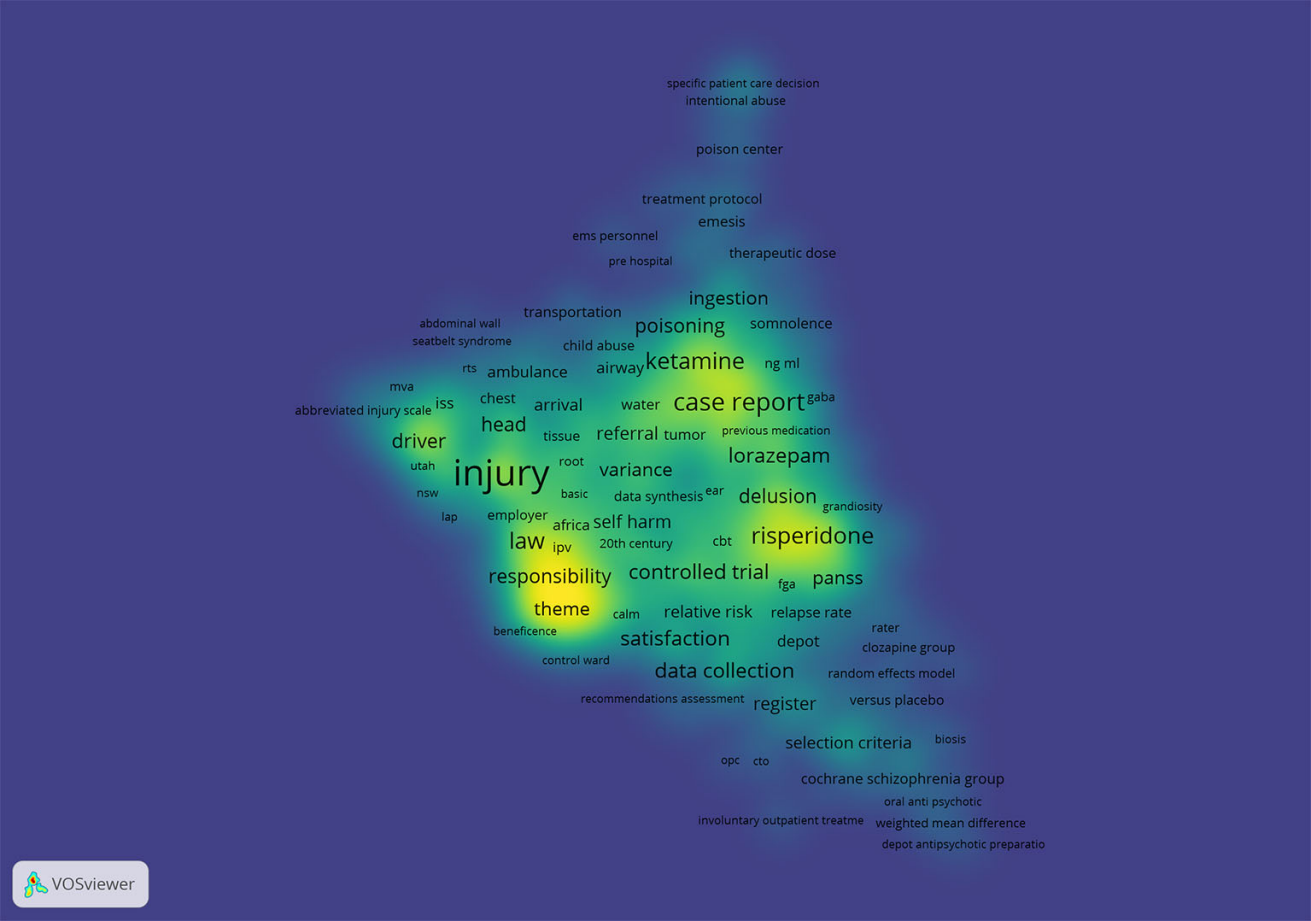
Visualization of similarities density visualization(39).

A dedicated search strategy combining text words and medical subject headings (MeSH) was composed for the MEDLINE database. Subsequently, a visualization of similarities (VOS) analysis was conducted using VOS viewer software (39) on the MEDLINE search results in order to identify candidate terms for notting out of the search strategy, to further specify the search. **Figure 1** shows the density visualization of the VOS analysis. Consequently, by scanning the resulting network, we excluded several search terms that would yield irrelevant studies for our search. The search strategy was adjusted by excluding the irrelevant terms. Exclusion of these irrelevant terms was checked in sensitivity searches. Subsequently, the MEDLINE search strategy was adjusted for the Embase and PsycINFO databases. The final search strategy is a sensitive compilation of terms describing the setting, the problem behavior that can cause the need for restraints and the types of restraints included, physical as well as pharmacological. For a comprehensive overview of the search terms used, we refer to the search strategy itself, added in **Supplementary File 1**. The final search will be repeated in the later stages of the systematic review to ensure the most recent publications will not be disregarded.

### Study Records

#### Data Management

The systematic review data management software application we will be using for eligibility screening of references is Rayyan (40). The search records will be checked for duplicates. Endnote will be used for bibliographic records management.

#### Selection Process

Two reviewers will take part in the selection process of studies. They will both screen all articles resulting from the search by title and abstract, independently from each other. Articles that appear to meet the inclusion criteria will be screened full text by each independent reviewer and will be reviewed for suitability using existing dedicated evaluation instruments for critically and systematically appraising articles. Also articles from which it is not clear if they will meet the inclusion criteria based on title and abstract screening will be screened full text by each independent reviewer and reviewed for suitability. Disagreements in the inclusion of articles arising from the title-abstract screening, as well as from the full text screening will be solved by discussion. If no agreement can be reached, a third independent researcher will be consulted to solve the disagreement. The review authors will not be blinded to any article information (such as journal names or author names).

#### Data Collection Process

A form to extract data from articles will be drawn up, tested, and used. We will elaborate on the data we will be extracting in *Data Items* section. The data extraction will be conducted by one reviewer, and verified by another reviewer. Disagreements will be sorted by discussion, and if no agreement can be reached a third independent researcher will be consulted. In case of uncertainties regarding the data or missing data the authors will be contacted *via* email for clarification or addition.

### Data Items

Data items we will be extracting include article information, such as year of publication, author(s), title, and journal name. We will also extract data on study characteristics, such as study design, e.g., observational studies or randomized controlled trials, and study setting, specifically the type of hospital ward, or an emergency department. Data will be extracted on the indications for restraint use, as well as data on the study interventions used, such as the use of physical restraint, or the use of pharmacological restraint. Subsequently, we will also include the study comparators used, such as no use of restraints, or the use of alternative measures. Furthermore, we will extract data on participant characteristics, such as average age, gender and the reason for admission to the hospital. We will also extract data on our outcomes of interest (**Table 1**). Finally, we will extract data on the type of sponsoring the studies had and their publication status.

### Outcomes and Prioritization

As mentioned before, in this section, we will elaborate on our outcomes of interest, as listed in **Table 1**. Starting with our primary outcomes of interest, for this review, we are interested in the effects of the use of physical or pharmacological restraints on the patient. We are interested in both beneficial and adverse effects of the use of restraints. We will firstly examine these effects using our primary outcome variables “length of hospital stay” and “complication rate”. The length of hospital stay can, depending on the value, imply a beneficial or an adverse effect. If there is a shorter length of hospital stay when using restraints compared to when not using restraints, this can imply a beneficial effect of the use of restraints, while if the length of hospital stay is longer an adverse effect of the restraint use might be implied. The length of stay will be measured in days. A higher complication rate also indicates adverse effects of the use of restraints. We intend to evaluate the overall complication rate, considering some studies will only report on the outcome complications, and will not distinguish the type of complications that have occurred. Additionally, we intend to differentiate according to type of complication for studies that do make a distinction. By surveying relevant literature, we found that complications can be arranged into several categories. Categories and respective tools of measurement include, cognitive performance, measured by, e.g., the Mini-Mental State Examination (MMSE) (41) or the Abbreviated Mental Test (AMT) (42, 43), behavioral problems, measured by, e.g., the Agitated Behavior Scale (ABS) (44), the Behavioral Activity Rating Scale (BARS) (45), the Richmond Agitation-Sedation Scale (RASS) (46), or the Confusion Assessment Method (CAM) (47), rate of falls, walking and other activities of Daily Living (ADL) dependence, measured, e.g., by the Modified Barthel Index (MBI) (48, 49), rate of pressure ulcers and rate of contractures (7). Articles tend to split the adverse effects in physical and mental health problems. We intend to use validated tools of measurement, if available.

Regarding the beneficial outcomes of restraint use, we are interested in the success rate of the restraint application, i.e., the percentage that the restraint has been effective, e.g., in controlling the behavior it was applied for. Two measures that are being used to specify this outcome are “time to tranquility or time to sedation,” as evaluated by different measurement tools, e.g., BARS, the ABS, or the RASS, and the need for additional restraints. The time (in minutes) until a certain threshold on the behavioral rating scale has been reached is assessed from the application of the restraint.

Secondarily, we are interested in the survival. This can give information on a beneficial or adverse effect of the use of physical or pharmacological restraints. For example, if the duration of survival of patients that were restrained at some point in the treatment is shorter than that of similar patients that were not restrained, it can indicate an adverse effect of the restraint.

Another secondary outcome of interest is symptom severity, split into physical and psychiatric symptoms. This will give information about the effects of the restraint on symptoms.

We are also interested in the effects of the use of physical or pharmacological restraints for the health care system in general. We are interested in the effects on health care providers who deal with applying restraints, such as physicians and nurses. We are also interested in the effect on the health costs. Moreover, we intend to review data on the cost effectiveness of the use of restraints.

### Risk of Bias in Individual Studies

The risk of bias will be assessed by one reviewer, and verified by another reviewer. Disagreements will be sorted by discussion, if no agreement can be reached a third independent researcher will be consulted. The risk of bias will be assessed by using dedicated tools such as the latest version of the Cochrane Collaboration Tool for assessing risk of bias (50), or the Newcastle-Ottawa scale (NOS) (51), depending on the type of articles included.

We will not exclude any articles according to risk of bias. Because of the small number of available articles expected on this subject, we want to include all available evidence. However, we will carefully document risk of bias and consider the risk of bias while interpreting the results.

### Data Synthesis

#### Criteria Under Which Data Will Be Quantitatively Synthesized

If studies are sufficiently homogeneous, a meta-analysis will be conducted on the collected data, using a random-effects model in the software program Comprehensive Meta-analysis. Whether or not a meta-analysis is possible will become apparent after data extraction. Also, if certain groups of studies are sufficiently homogeneous, due to the diversity of outcomes, we are including, we will conduct a meta-analysis on part of the included studies.

#### Planned Methods for Summarization, Handling Data and Combining Data

We aim at comparing the different interventions and comparators listed in *Data Items* section, depending upon study availability. We will compare restraints with all available comparators including no intervention. This implies that, if two different forms of restraints are compared, we will compare these restraints to investigate their beneficial and adverse effects, e.g., benzodiazepines vs. antipsychotics. We will summarize all extracted data using tables and where possible graphs. If we come across missing data, we will contact the study authors to attempt to obtain the missing data. Assessment of heterogeneity will take place using relevant tests.

#### Proposed Additional Analysis

If deemed relevant, meta-regression analysis can be used to explain differences in outcomes between studies and subgroups.

#### Type of Summary Planned If Quantitative Synthesis Is Not Appropriate

If the data or part of the data is not suitable to conduct quantitative tests, those results will be presented in a narrative form using text and tables. The resulting systematic narrative synthesis will provide an overview of the characteristics and results of the included studies using tables. It will also provide a narrative comparison of the findings of the different studies, thereby finding similarities and differences between the studies.

### Meta-Bias(es)

To determine whether outcome reporting bias is present, we will check as to whether selective reporting of outcomes is present in included studies by screening protocols in the International Clinical Trials Registry Platform (ICTRP) of the World Health Organization (WHO) (52). If no protocol is available we will compare the method section to the results section. We will also have to take into account selection bias, considering we will include observational non-randomized studies, which are especially susceptible to selection bias. Since non-randomized studies are also susceptible to performance bias and detection bias, we will also check for these biases. In case there are withdrawals from studies, we will check for attrition bias. We will also check for publication bias and small sample bias where possible.

### Confidence in Cumulative Evidence

The strength of the body of gathered evidence will be weighed according to the GRADE methodology (Grading Recommendations Assessment, Development and Evaluation) (53).

## Discussion

This project aims to systematically review the available literature on the beneficial and adverse effects of the use of physical and pharmacological restraints in hospital care. Our objective is to assemble the available evidence on this subject, and to subsequently provide a critical assessment of the gathered evidence. Results of our review may more optimally guide health professionals in their choice when to refrain from applying restraints in the hospital and to use restraints in a correct way. Additionally, the outcome of our review might aid health professionals in properly discussing the subject of restraints with patients and relatives. Moreover, results will identify knowledge gaps that need to be targeted in order to bring about changes to the daily clinical practice of restraints.

In nursing homes and other non-hospital settings, there have been gradual advances in limiting the use of restraints. For these settings, guidelines and literature overviews have been synthesized and implemented (54–62). For example, in nursing homes throughout the United States, there have already been positive results in reducing the use of restraints, limiting the use of restraints in nursing homes from 40% in the 1980s to 10% in 2008 (7). While restraint use in nursing homes may be reduced even further, these results hold promise for the reduction of restraints in the hospital setting. Moreover, we will not address the use of restraints in psychiatric care facilities. These restraints are usually being applied under a different legal system, which makes it difficult to compare outcomes. Nevertheless, restraints used on psychiatric patients in general hospitals (excluding the psychiatric ward) will be included in the current review.

One of the limitations inherent to all systematic reviews is the amount of evidence available. In our scoping search, we encountered a small amount of studies that focus specifically on the hospital setting. Effects of restraints in the nursing home setting have been better studied, but it remains unknown as to what extent this evidence translates to the hospital setting (28, 30, 63). For the psychiatric setting, evidence on the effects of restraints is also available, although reviews by Nelstrop and Sailas stated the lack of experimental studies as an important limitation (29, 64, 65). Both the patient population (including, e.g., mental and physical health) and the setting (e.g., the amount and qualifications of staff, the architecture of the ward) may cause differences in the effects of restraints in the hospital vs. the nursing home setting. Reviewing the available evidence for the hospital setting therefore seems important to provide health professionals in hospitals with clinically relevant effect estimates regarding the use of restraints. Moreover, the quality of the evidence may limit the conclusions that can be drawn. Given the expected lack of RCTs, residual confounding or reverse causation may induce biases. Nevertheless, conclusions form the best available evidence may inspire future (non-inferiority) RCTs on the effects of restraints in the hospital.

In our scoping search on pharmacological, or chemical, restraints, we found that these terms are used rather sparsely to identify relevant literature. Moreover, we found that not all studies on pharmacological restraints use either the term pharmacological or chemical restraint. This proposed difficulties in composing a comprehensive, but not too broad, search strategy. By additionally including search terms on specific psychopharmacological interventions that may be used as chemical restraint (e.g., benzodiazepines, antipsychotics, and opioids), we aimed at including all relevant evidence. Another difficulty we encountered lies in the definition of pharmacological restraint that we use. Namely, in the introduction, we stated that the pharmaceutical products used as pharmacological restraints cannot be used to treat a medical condition. However, the distinction between treatment of a medical condition and use as a restraint will not always be clear. For example, when treating a psychotic disorder accompanied by aggressive behavior with an antipsychotic drug, the aim of the treatment can be twofold. On the one hand, the aim is to treat the psychosis, and on the other hand, the sedative effect the antipsychotic has can reduce the aggressive behavior. We will critically examine whether this distinction is clear in the studies we include, and take this into account when interpreting the data. Nevertheless, this difficulty in differentiation between treatment vs. chemical restraint remains a potential limitation of this study.

We will not specifically address measures that are used as an alternative to restraints on their own. Nevertheless, we will include all studies that compare the effects of physical or chemical restraints to specific alternatives that consist of domotics (home automation) or psychological interventions. Another example of such an alternative measure is the presence of a relative to calm a disquieted patient, also called “rooming in.” Considering the idea that benefits of restraints might not outweigh their adverse effects, these measures might provide a viable alternative. Results of our systematic search may determine as to which extent it is more beneficial and safer to apply these interventions instead of restraints, in which the experiences of both patients and their relatives are important (10, 66–68).

A topic that will not be addressed by this systematic review is the ethical aspect of applying restraints. Cultural differences in the view on restraints may influence the way the beneficial and adverse effects of restraints are being evaluated. Moreover, political forces may impose clinical decisions that are not in line with available evidence. Nevertheless, outcomes of the current review may form an important contribution to the (ethical) debate on the application of restraints in the hospital.

Regarding dissemination of the results of our systematic review, we plan to submit results for publication in peer reviewed scientific journals. Moreover, we intent to present findings at meetings of relevant scientific societies. In addition, literature databases and curated extracted data will be available for interested collaborators on request. Finally, we will write a report for the funding organization, as commissioned by the Dutch Ministry of Health, Welfare and Sport.

In conclusion, with this systematic review protocol, we describe the methodology of our systematic search on the beneficial and adverse effects of physical and chemical restraints in the hospital. We hope that this review will provide health professionals with evidence based knowledge to better guide their choice to apply or refrain from restraints. If the notion is correct, in that for most indications negative effects of restraints outweigh their positive effect, this may lead to a reduction in the use of restraints in the hospital, as has already been achieved in nursing homes. In addition, by addressing knowledge gaps, this review will direct future research that is needed to improve the clinical application of restraints. Moreover, the outcomes of our systematic review can be used in the composition of a multidisciplinary guideline.

## Author Contributions

WB and RM drafted the first versions of the protocol and manuscript, all authors critically reviewed the protocol and the manuscript and provided comments on the drafts. WB processed these comments. RM and WB provided for the registration of this protocol in Prospero. WB, RM, and JD drafted the search strategy.

## Funding

The study is funded by ZonMW, a Dutch independent financing organization for health research and innovation. The funding source had no role in the design of this study and will not have any role during its execution, analyses, interpretation of the data, or decision to submit results.

## Conflict of Interest

The authors declare that the research was conducted in the absence of any commercial or financial relationships that could be construed as a potential conflict of interest.

## Abbreviations

ADL, activities of daily living; AMT, Abbreviated Mental Test; ABS, Agitated Behavior Scale; BARS, Behavioral Activity Rating Scale; CAM, Confusion Assessment Method; GRADE, Grading Recommendations Assessment, Development and Evaluation; ICTRP, International Clinical Trials Registry Platform; MBI, Modified Barthel Index; MeSH, medical subject headings; MMSE, Mini-Mental State Examination; NOS, Newcastle-Ottawa Scale; PRISMA, preferred reporting items for systematic review and meta-analysis; PRISMA-P, preferred reporting items for systematic review and meta-analysis protocols; RASS, Richmond Agitation-Sedation Scale; VOS, visualization of similarities; WHO, World Health Organization.
