# Supplementary material for: Physical and Pharmacological Restraints in Hospital Care: Protocol for a Systematic Review
Source: Front Psychiatry. 2020 Feb 28;10:921. doi: 10.3389/fpsyt.2019.00921 (PMC7058582; doi:10.3389/fpsyt.2019.00921)
Supplement: Supplementary file 1 [file DataSheet_1.pdf]

	**Ovid MEDLINE(R) and Epub Ahead of Print, In-Process & Other Non-Indexed Citations, and Daily <1946 to July 30, 2018>**<br>**Search date: 31 July 2018**	
**#**	**Searches**	**Results**
1	exp hospital units/ or exp hospitals/ or exp hospital departments/ or exp hospitalization/ or "hospital, personnel"/ or "attitude of health personnel"/	739471
2	(hospital* or icu or intensive care unit? or emergency department? or (emergency adj2 (care or center? or service?)) or doctor? or (nurse? not (nursing home? or nursing facilit* or long term care facilit* or residential care facilit* or "homes for the aged"))).ab,jw,kf,ti.	1718433
3	or/1-2 [in hospital]	2000649
4	restraint?.mp.	28912
5	3 and 4	3527
6	(coercion or coercive).ab,kf,sh,ti.	7969
7	problem behavior/ or aggression/ or hostility/ or "irritable mood"/ or "psychomotor agitation"/ or "dangerous behavior"/	45141
8	((problem* or abusive or excessive or inappropriate or uninhibited or impairment or hostil* or irritab* or obstreperous or uncooperative or non compliant) adj2 behav*).ab,kf,ti.	26044
9	(aggression or agitation or calling or disinhibition or demanding attention or hostility or hyperactiv* or irritability or labil* or nocturnal unrest or rebell* or recalcitrant or pacing or psychotic or psychosis or "Rejection of care" or Repetitive questioning or Resistiveness to care or Restlessness or Restiveness or insomnia or sleep disorder? or sleep deprivat* or violen* or (property adj 2 damag*) or disorient* or dangerousness or unrest or wandering or cursing or swearing or Negativism or grabbing or noise? or sun down* or sundown* or endanger* or (patient? adj1 violen*)).ab,kf,ti.	467314
10	(Inappropriate robbing or disrobing or undress* or Hitting or Screaming or Hoarding or Scratching or self inflict* or suicid* or automutil* or Hurting or Tearing or Throwing or Biting or Eating or sexual advances or sexual advances or Intentional falling or Aberrant motor behavior or Night time disturbances or threatening).ab,kf,ti.	243814
11	or/7-10 [problem behavior]	734968
12	and/3,6,11	362

13	(Sensor mat or floor mat alarm or pressure-sensitive mats or sensor pad or Motion sensor or motion detector or Low bed or low-low bed or low height beds or high-low beds or low-rise beds or Bed alarm or chair alarm or bed exit alarms or wheelchair alarm or bedside infrared beam detectors or in-bed pressure sensors or (fall adj2 (prevention monitor or system or alarm)) or posey alarm or magnetic safety alarm or bedside alarm or seatbelt alarm or safety alarm or patient safety alarm or room alarm or door alarm or exit alarm or wander alarm or Half length side rails or bed rails or Ball blanket or weighted blanket or therapy blanket or heavy blanket or Padded walls or padded cell or fall mat or padded mat or impact-absorbing walls or impact absorbing floors or Foam Bumpers or safety guard rail or rail bumper pad or Hip protector pants or padded clothing or hip pad or hip protector or Acoustical surveillance or nuisance alarms or fall detection system or fall detection device or Acoustic Fall Detection or Acoustic fall Monitoring or automatic fall detection or Ball chair or Protac SenSit or sensory-stimulating chair or Sensory Chairs or sensory seating or Sensory-Friendly Chairs or multi-sensory furniture or Sensory Therapeutic Seating or Bean Bag Chairs or wander detection or wandering detection or wandering prevention or Immobilizer or elbow-immobilizer or freedom sleeves or soft splints or secure sleeve or global positioning system or Camera surveillance or video surveillance or Camera-Based Fall Detection or Safety pillow or bed roll or Fall Safety Cushions or Elderly Safety Pads or bolster cushions or perimeter cover or Positioning Cushions or Positioning pillow or roll guards or roll cushion or roll pillow or positioning roll or soft rails or rail pad or rail cover or Mittens or restraining mitts or hand restraint or posey mitts or control mitts or secure mitts or Wedge pillow or Foam Wedges or rail wedges or Wander detection sensor or Television timer Side rails or bed rails or Protective helmet or Enclosure bed or cage bed or posey bed or bed box or Wrist restraint or ankle restraint or foot restraint or hand restraint or hand holder or foot holder or Limb restraint or soft limb restraints or locked cuff restraints or limb holder or arm restraint or leg restraint or Cushion belt or abdominal restraint or Vest restraint or Posey vest or safety vest or safety suit or safety harnesses or trunk restraint or chest restraint or roll belt or body holder or roll jacket or Wheelchair restraint or chair restraint or lap belt or wheelchair belt or chair belt or seatbelt or lap restraint or crotch ties or pelvic ties or soft belt or padded belt or pelvic holder or Chair with locked table or overchair tables or chairs prevent rising or Gerichairs or geriatric chairs with fixed table or recliner chairs with fixed table or chairs with fixed tray table or constricting chair or Blanket restraint or sheet ties or full sheet restraint).ab,kf,ti. [other physical restraints]	4653
14	3 and 13	561
15	central nervous system depressants/ or exp "anesthetics, general"/ or exp narcotics/ or exp tranquilizing agents/ or "hypnotics and sedatives"/	406066
16	(restrain* or immobil*).ab,kf,ti.	151627
17	15 and 16	4140
18	(antipsychot* or anti psychot* or benzodiazep*).ab,kf,ti.	67726
19	ketamine/	11167

20	(ketamin* or anesject or Brevinaze or calipsol or calypsol or imalgene or Ivanes or kalipsol or Kanox or katamine or Keiran or Ketacor or keta-hameln or ketaject or ketalar or ketalin or Ketalor or ketamax or ketaminol vet or ketanest or ketased or ketaset or Ketashort or Ketava or ketaved or ketavet or Ketazol or ketmin or ketoject or ketolar or narkamon or narketan or special k or soon-soon or tekam or velonarcon or vetalar).ab,kf,ti.	16703
21	"6740-88-1".rn.	0
22	propranolol/	31824
23	(propranolol* or Angilol or Bedranol or Cardinol or Ciplar or Deralin or Dociton or Duranol or Inderal or Indobloc or INNOPRAN or Prophylux or Sumial or Propanolol or Avlocardyl or AY-20694 or AY 20694 or AY20694 or Rexigen or Dexpropranolol or Obsidan or Obzidan or Anaprilin or Anapriline or Betadren).ab,kf,ti.	33080
24	"525-66-6".rn.	0
25	or/17-24	132280
26	animals/ not humans/	4446437
27	25 not 26 [chemical restraints]	84965
28	and/3,11,27	2879
29	or/5,12,14,28	6978
30	(Motor vehicle or seatbelt or Booster seat or road user or male driver or frontal crash or airbag deployment or seabelt legislation or child passenger or vehicle occupant or road traffic collision or ejection or Child safety seat or child passenger safety or safe transportation or driving or road traffic injury or road traffic crash or automobile or passenger or vehicle type or mph or airbag or steering wheel or car or mva? or veterinary or preterm infant or premature infant or cat or fetus or birth weight or neonate or road safety or back seat or cyclist or driver or motorcyclist or mvc).ab,kf,ti. [VOS yellow]	488217
31	(acinetobacter baumannii or extracorporeal membrane oxygen or police custody or bacterium or munchausen syndrome or rape or school nurse or public health nurse or partner violence or fiscal restraint? or financial restraint? or prison or detention or reproductive coercion or psychiatric ward or psychiatric inpatient unit or psychiatric inpatient treatment).ab,kf,ti. [VOS red]	104471
32	(Cochrane schizophrenia group or Emergency psychiatric service).ab,kf,ti. [VOS purple]	193
33	(Dietary restraint or schizophrenic outpatient or psychiatric outpatient clinic or eating disorder?).ab,kf,ti. [VOS green]	18205
34	(child abuse or activated charcoal or charcoal).ab,kf,ti. [VOS light blue]	19238
35	(antimicrobial or gastric lavage or animal? or antidote or poison*).ab,kf,ti. [VOS dark blue]	1210661
36	or/30-35	1785719
37	29 not 36	5879
38	remove duplicates from 37	5866

	**Ovid Embase Classic+Embase <1947 to 2018 July 30>**<br>**Search date: 31 July 2018**	
**#**	**Searches**	**Results**

1	*hospital/ or *community hospital/ or *general hospital/ or *geriatric hospital/ or *"exp hospital subdivisions and components"/ or *non profit hospital/ or *pediatric hospital/ or *private hospital/ or *public hospital/ or *exp teaching hospital/ or *hospitalization/ or *hospital personnel/ or *hospital physician/ or *medical staff/ or *resident/ or *nurse attitude/ or *physician assistant attitude/ or *physician attitude/ or *hospital discharge/ or hospital management/ or *hospital admission/ or *hospital readmission/	291475
2	(hospital* or icu or intensive care unit? or emergency department? or (emergency adj2 (care or center? or service?)) or doctor? or (nurse? not (nursing home? or nursing facilit* or long term care facilit* or residential care facilit* or "homes for the aged"))).ab,jx,kw,ti.	2440225
3	or/1-2 [in hospital]	2532389
4	restraint?.ab,hw,kw,ti.	29130
5	3 and 4	3484
6	(coercion or coercive).ab,kw,ti.	5494
7	*aggression/ or *hostility/ or *aggresiveness/ or *irritability/ or *restlessness/ or "dangerous behavior"/	68708
8	((problem* or abusive or excessive or inappropriate or uninhibited or impairment or hostil* or irritab* or obstreperous or uncooperative or non compliant) adj2 behav*).ab,kw,ti.	33842
9	(aggression or agitation or calling or disinhibition or demanding attention or hostility or hyperactiv* or irritability or labil* or nocturnal unrest or rebell* or recalcitrant or pacing or psychotic or psychosis or "Rejection of care" or Repetitive questioning or Resistiveness to care or Restlessness or Restiveness or insomnia or sleep disorder? or sleep deprivat* or violen* or (property adj 2 damag*) or disorient* or dangerousness or unrest or wandering or cursing or swearing or Negativism or grabbing or noise? or sun down* or sundown* or endanger* or (patient? adj1 violen*)).ab,kw,ti.	625625
10	(Inappropriate robbing or disrobing or undress* or Hitting or Screaming or Hoarding or Scratching or self inflict* or suicid* or automutil* or Hurting or Tearing or Throwing or Biting or Eating or sexual advances or sexual advances or Intentional falling or Aberrant motor behavior or Night time disturbances or threatening).ab,kw,ti.	332781
11	or/7-10 [problem behavior]	981128
12	and/3,6,11	327

13	(Sensor mat or floor mat alarm or pressure-sensitive mats or sensor pad or Motion sensor or motion detector or Low bed or low-low bed or low height beds or high-low beds or low-rise beds or Bed alarm or chair alarm or bed exit alarms or wheelchair alarm or bedside infrared beam detectors or in-bed pressure sensors or (fall adj2 (prevention monitor or system or alarm)) or posey alarm or magnetic safety alarm or bedside alarm or seatbelt alarm or safety alarm or patient safety alarm or room alarm or door alarm or exit alarm or wander alarm or Half length side rails or bed rails or Ball blanket or weighted blanket or therapy blanket or heavy blanket or Padded walls or padded cell or fall mat or padded mat or impact-absorbing walls or impact absorbing floors or Foam Bumpers or safety guard rail or rail bumper pad or Hip protector pants or padded clothing or hip pad or hip protector or Acoustical surveillance or nuisance alarms or fall detection system or fall detection device or Acoustic Fall Detection or Acoustic fall Monitoring or automatic fall detection or Ball chair or Protac SenSit or sensory-stimulating chair or Sensory Chairs or sensory seating or Sensory-Friendly Chairs or multi-sensory furniture or Sensory Therapeutic Seating or Bean Bag Chairs or wander detection or wandering detection or wandering prevention or Immobilizer or elbow-immobilizer or freedom sleeves or soft splints or secure sleeve or global positioning system or Camera surveillance or video surveillance or Camera-Based Fall Detection or Safety pillow or bed roll or Fall Safety Cushions or Elderly Safety Pads or bolster cushions or perimeter cover or Positioning Cushions or Positioning pillow or roll guards or roll cushion or roll pillow or positioning roll or soft rails or rail pad or rail cover or Mittens or restraining mitts or hand restraint or posey mitts or control mitts or secure mitts or Wedge pillow or Foam Wedges or rail wedges or Wander detection sensor or Television timer Side rails or bed rails or Protective helmet or Enclosure bed or cage bed or posey bed or bed box or Wrist restraint or ankle restraint or foot restraint or hand restraint or hand holder or foot holder or Limb restraint or soft limb restraints or locked cuff restraints or limb holder or arm restraint or leg restraint or Cushion belt or abdominal restraint or Vest restraint or Posey vest or safety vest or safety suit or safety harnesses or trunk restraint or chest restraint or roll belt or body holder or roll jacket or Wheelchair restraint or chair restraint or lap belt or wheelchair belt or chair belt or seatbelt or lap restraint or crotch ties or pelvic ties or soft belt or padded belt or pelvic holder or Chair with locked table or overchair tables or chairs prevent rising or Gerichairs or geriatric chairs with fixed table or recliner chairs with fixed table or chairs with fixed tray table or constricting chair or Blanket restraint or sheet ties or full sheet restraint).ab,kw,ti. [other physical restraints]	5234
14	3 and 13	725
15	*central depressant agent/ or *anestetic agent/ or *narcotic agent/ or *tranquilizer/ or *hypnotic sedative agent/	19000
16	(restrain* or immobil*).ab,kw,ti.	191575
17	15 and 16	232
18	(antipsychot* or anti psychot* or benzodiazep*).ab,kw,ti.	102572
19	*ketamine/	15803

20	(ketamin* or anesject or Brevinaze or calipsol or calypsol or imalgene or Ivanes or kalipsol or Kanox or katamine or Keiran or Ketacor or keta-hameln or ketaject or ketalar or ketalin or Ketalor or ketamax or ketaminol vet or ketanest or ketased or ketaset or Ketashort or Ketava or ketaved or ketavet or Ketazol or ketmin or ketoject or ketolar or narkamon or narketan or special k or soon-soon or tekam or velonarcon or vetalar).ab,kw,ti,tn.	24941
21	"6740-88-1".rn.	31956
22	*propranolol/	51784
23	(propranolol* or Angilol or Bedranol or Cardinol or Ciplar or Deralin or Dociton or Duranol or Inderal or Indobloc or INNOPRAN or Prophylux or Sumial or Propanolol or Avlocardyl or AY-20694 or AY 20694 or AY20694 or Rexigen or Dexpropranolol or Obsidan or Obzidan or Anaprilin or Anapriline or Betadren).ab,kw,ti,tn.	47206
24	"525-66-6".rn.	92865
25	or/17-24	238977
26	(animal/ or animal experiment/ or animal model/ or nonhuman/ or rat/ or mouse/ or (rat or rats or mouse or mice).ti.) not human/	6447403
27	25 not 26 [chemical restraints]	172045
28	and/3,11,27	6128
29	or/5,12,14,28	10275
30	(Motor vehicle or seatbelt or Booster seat or road user or male driver or frontal crash or airbag deployment or seabelt legislation or child passenger or vehicle occupant or road traffic collision or ejection or Child safety seat or child passenger safety or safe transportation or driving or road traffic injury or road traffic crash or automobile or passenger or vehicle type or mph or airbag or steering wheel or car or mva? or veterinary or preterm infant or premature infant or cat or fetus or birth weight or neonate or road safety or back seat or cyclist or driver or motorcyclist or mvc).ab,kw,ti. [VOS yellow]	682355
31	(acinetobacter baumannii or extracorporeal membrane oxygen or police custody or bacterium or munchausen syndrome or rape or school nurse or public health nurse or partner violence or fiscal restraint? or financial restraint? or prison or detention or reproductive coercion or psychiatric ward or psychiatric inpatient unit or psychiatric inpatient treatment).ab,kw,ti. [VOS red]	119603
32	(Cochrane schizophrenia group or Emergency psychiatric service).ab,kw,ti. [VOS purple]	283
33	(Dietary restraint or schizophrenic outpatient or psychiatric outpatient clinic or eating disorder?).ab,kw,ti. [VOS green]	25034
34	(child abuse or activated charcoal or charcoal).ab,kw,ti. [VOS light blue]	25919
35	(antimicrobial or gastric lavage or animal? or antidote or poison*).ab,kw,ti. [VOS dark blue]	1609870
36	or/30-35	2383558
37	29 not 36	8630
38	(canadian or elsevier or embase).cr.	25507781
39	37 and 38	7140

	**Ovid PsycINFO 1806 to July Week 4 2018**<br>**Search date 31 July 2018**	
**#**	**Searches**	**Results**
1	hospitals/ or hospitalization/ or hospital admission/ or hospital discharge/ or physicians/	42806
2	(hospital* or icu or intensive care unit? or emergency department? or (emergency adj2 (care or center? or service?)) or doctor? or (nurse? not (nursing home? or nursing facilit* or long term care facilit* or residential care facilit* or "homes for the aged"))).ab,jx,id,ti.	224339
3	or/1-2 [in hospital]	237231
4	restraint?.ab,hw,id,ti.	11942
5	3 and 4	1298
6	(coercion or coercive).ab,id,ti.	7729
7	aggressive behavior/ or aggressiveness/ or patient violence/ or hostility/ or irritability/ or restlessness/ or dangerousness/	34630
8	((problem* or abusive or excessive or inappropriate or uninhibited or impairment or hostil* or irritab* or obstreperous or uncooperative or non compliant) adj2 behav*).ab,id,ti.	50621
9	(aggression or agitation or calling or disinhibition or demanding attention or hostility or hyperactiv* or irritability or labil* or nocturnal unrest or rebell* or recalcitrant or pacing or psychotic or psychosis or "Rejection of care" or Repetitive questioning or Resistiveness to care or Restlessness or Restiveness or insomnia or sleep disorder? or sleep deprivat* or violen* or (property adj 2 damag*) or disorient* or dangerousness or unrest or wandering or cursing or swearing or Negativism or grabbing or noise? or sun down* or sundown* or endanger* or (patient? adj1 violen*)).ab,id,ti.	309074
10	(Inappropriate robbing or disrobing or undress* or Hitting or Screaming or Hoarding or Scratching or self inflict* or suicid* or automutil* or Hurting or Tearing or Throwing or Biting or Eating or sexual advances or sexual advances or Intentional falling or Aberrant motor behavior or Night time disturbances or threatening).ab,id,ti.	126475
11	or/7-10 [problem behavior]	464305
12	and/3,6,11	238

13	(Sensor mat or floor mat alarm or pressure-sensitive mats or sensor pad or Motion sensor or motion detector or Low bed or low-low bed or low height beds or high-low beds or low-rise beds or Bed alarm or chair alarm or bed exit alarms or wheelchair alarm or bedside infrared beam detectors or in-bed pressure sensors or (fall adj2 (prevention monitor or system or alarm)) or posey alarm or magnetic safety alarm or bedside alarm or seatbelt alarm or safety alarm or patient safety alarm or room alarm or door alarm or exit alarm or wander alarm or Half length side rails or bed rails or Ball blanket or weighted blanket or therapy blanket or heavy blanket or Padded walls or padded cell or fall mat or padded mat or impact-absorbing walls or impact absorbing floors or Foam Bumpers or safety guard rail or rail bumper pad or Hip protector pants or padded clothing or hip pad or hip protector or Acoustical surveillance or nuisance alarms or fall detection system or fall detection device or Acoustic Fall Detection or Acoustic fall Monitoring or automatic fall detection or Ball chair or Protac SenSit or sensory-stimulating chair or Sensory Chairs or sensory seating or Sensory-Friendly Chairs or multi-sensory furniture or Sensory Therapeutic Seating or Bean Bag Chairs or wander detection or wandering detection or wandering prevention or Immobilizer or elbow-immobilizer or freedom sleeves or soft splints or secure sleeve or global positioning system or Camera surveillance or video surveillance or Camera-Based Fall Detection or Safety pillow or bed roll or Fall Safety Cushions or Elderly Safety Pads or bolster cushions or perimeter cover or Positioning Cushions or Positioning pillow or roll guards or roll cushion or roll pillow or positioning roll or soft rails or rail pad or rail cover or Mittens or restraining mitts or hand restraint or posey mitts or control mitts or secure mitts or Wedge pillow or Foam Wedges or rail wedges or Wander detection sensor or Television timer Side rails or bed rails or Protective helmet or Enclosure bed or cage bed or posey bed or bed box or Wrist restraint or ankle restraint or foot restraint or hand restraint or hand holder or foot holder or Limb restraint or soft limb restraints or locked cuff restraints or limb holder or arm restraint or leg restraint or Cushion belt or abdominal restraint or Vest restraint or Posey vest or safety vest or safety suit or safety harnesses or trunk restraint or chest restraint or roll belt or body holder or roll jacket or Wheelchair restraint or chair restraint or lap belt or wheelchair belt or chair belt or seatbelt or lap restraint or crotch ties or pelvic ties or soft belt or padded belt or pelvic holder or Chair with locked table or overchair tables or chairs prevent rising or Gerichairs or geriatric chairs with fixed table or recliner chairs with fixed table or chairs with fixed tray table or constricting chair or Blanket restraint or sheet ties or full sheet restraint).ab,id,ti. [other physical restraints]	1088
14	3 and 13	82
15	cns depressant drugs/ or anesthetic drugs/ or narcotic drugs/ or tranquilizing drugs/	5373
16	(restrain* or immobil*).ab,id,ti.	20704
17	15 and 16	138
18	(antipsychot* or anti psychot* or benzodiazep*).ab,id,ti.	37664
19	ketamine/	1689

20	(ketamin* or anesject or Brevinaze or calipsol or calypsol or imalgene or Ivanes or kalipsol or Kanox or katamine or Keiran or Ketacor or keta-hameln or ketaject or ketalar or ketalin or Ketalor or ketamax or ketaminol vet or ketanest or ketased or ketaset or Ketashort or Ketava or ketaved or ketavet or Ketazol or ketmin or ketoject or ketolar or narkamon or narketan or special k or soon-soon or tekam or velonarcon or vetalar).ab,id,ti.	3004
21	propranolol/	887
22	(propranolol* or Angilol or Bedranol or Cardinol or Ciplar or Deralin or Dociton or Duranol or Inderal or Indobloc or INNOPRAN or Prophylux or Sumial or Propanolol or Avlocardyl or AY-20694 or AY 20694 or AY20694 or Rexigen or Dexpropranolol or Obsidan or Obzidan or Anaprilin or Anapriline or Betadren).ab,id,ti.	1925
23	or/17-22	42358
24	limit 23 to human [chemical restraints]	33161
25	and/3,11,24	1864
26	or/5,12,14,25	3367
27	(Motor vehicle or seatbelt or Booster seat or road user or male driver or frontal crash or airbag deployment or seabelt legislation or child passenger or vehicle occupant or road traffic collision or ejection or Child safety seat or child passenger safety or safe transportation or driving or road traffic injury or road traffic crash or automobile or passenger or vehicle type or mph or airbag or steering wheel or car or mva? or veterinary or preterm infant or premature infant or cat or fetus or birth weight or neonate or road safety or back seat or cyclist or driver or motorcyclist or mvc).ab,id,ti. [VOS yellow]	63887
28	(acinetobacter baumannii or extracorporeal membrane oxygen or police custody or bacterium or munchausen syndrome or rape or school nurse or public health nurse or partner violence or fiscal restraint? or financial restraint? or prison or detention or reproductive coercion or psychiatric ward or psychiatric inpatient unit or psychiatric inpatient treatment).ab,id,ti. [VOS red]	36874
29	(Cochrane schizophrenia group or Emergency psychiatric service).ab,id,ti. [VOS purple]	91
30	(Dietary restraint or schizophrenic outpatient or psychiatric outpatient clinic or eating disorder?).ab,id,ti. [VOS green]	23823
31	(child abuse or activated charcoal or charcoal).ab,id,ti. [VOS light blue]	11963
32	(antimicrobial or gastric lavage or animal? or antidote or poison*).ab,id,ti. [VOS dark blue]	141163
33	or/27-32	271634
34	26 not 33	3051
